# Nerve transfer surgery in spinal cord injury: online information sharing

**DOI:** 10.1186/s12883-021-02209-5

**Published:** 2021-04-24

**Authors:** Syena Moltaji, Christine B. Novak, Jana Dengler

**Affiliations:** 1 Division of Plastic and Reconstructive Surgery, Department of Surgery, University of Toronto, Toronto, ON Canada; 2 Division of Plastic and Reconstructive Surgery, The Hospital for Sick Children, Toronto, ON Canada; 3 Division of Plastic and Reconstructive Surgery, Tory Trauma Program, Sunnybrook Health Sciences Centre, Toronto, ON Canada

**Keywords:** Spinal cord injuries, Tetraplegia, Nerve transfer, Upper extremity, Information seeking behavior, Information dissemination, Social media, Online communities

## Abstract

**Background:**

Nerve transfer to improve upper extremity function in persons with cervical spinal cord injury (SCI) is a new reconstructive option, and has led to more people seeking and sharing surgical information and experiences. This study evaluated the role of social media in information-sharing on nerve transfer surgery within the SCI community.

**Methods:**

Data were collected from Facebook, which is the favored information-sharing platform among individuals seeking medical information. Searched terms included ‘spinal cord injury’ and ‘SCI’ and excluded groups with: less than two members (*n* = 7); closed groups (*n* = 2); not pertaining to SCI (*n* = 13); restricted access (*n* = 36); and non-English (n = 2). Within public and private accessed groups, searches were conducted for ‘nerve’, ‘transfer’, ‘nerve transfer’, and ‘nerve surgery’. Each post about nerve transfer, responses to posts, and comments about nerve transfer in response to unrelated posts were tabulated. Thematic content analyses were performed and data were categorized as seeking information, sharing information, sharing support, and sharing appreciation.

**Results:**

The search yielded 99 groups; 35 met the inclusion criteria (average size = 2007, largest = 12,277). Nerve transfer was discussed in nine groups, with 577 total mentions. In the seeking information axis, posts were related to personal experience (54%), objective information (31%), surgeon/center performing the procedure (9%), and second opinion (4%). At least 13% of posts were from individuals learning about nerve transfers for the first time. In the sharing information axis, the posts: shared personal experience (52%); shared objective information (13%); described alternative treatment (3%); tagged someone to share information (11%); linked to outside resources (12%); and recommended a specific surgeon/center (9%).

**Conclusion:**

Social media is an important source of information and support for people with SCI. There is a paucity of information on nerve transfers. These study findings will inform implementation of future education strategies.

## Background

Spinal cord injury (SCI) is a devastating event with a lifelong impact. Cervical SCI specifically compromises upper extremity function integral to activities of daily living and self-care [1–3]. The emotional, financial, and interpersonal impact of cervical SCI on individuals and their caregivers makes regaining motor function imperative [1, 4]. Improvement of upper extremity function has been identified as the highest patient-reported priority for improving quality of life [5, 6]. Nerve transfer surgery has emerged as a promising opportunity to improve upper extremity function in cervical SCI, and may be a viable treatment option for individuals in whom other treatments are not available [7–10]. Although studies have shown significant gains in upper extremity range of motion and strength after nerve transfer [11, 12], use of upper extremity reconstruction remains low around the world [13, 14].

One barrier which may contribute to low utilization rates is lack of awareness in the SCI community of surgical options to improve upper extremity function [15]. To address this lack of knowledge, it is important to identify where individuals seek information on treatment options, and what information is available. One study found a paucity of upper extremity reconstruction information available on the internet [16]. This study did not investigate the involvement of social media as a source of support and information. Individuals utilize social media to obtain a better understanding of a breadth of health conditions [17–21]. For example, there are over 2.5 billion users on Facebook, and the groups that collect on this platform create a powerful forum for knowledge exchange [17–21]. Social groups and online communities have been shown to be an important source of support for individuals with SCI and their caregivers [4, 22]. The education role of these online SCI groups has not been elucidated. The purpose of this study was to evaluate the use of social media in information-sharing within the SCI community, and specifically to review the discussion of nerve transfer to better understand the role of online SCI communities in education and decision-making.

## Methods

### Search strategy

Data were collected from the online social media network, Facebook, which is the favored information-sharing platform among health communities [17]. The group function of Facebook was searched with the terms ‘SCI’, ‘spinal cord injury’, ‘tetraplegia’, ‘tetraplegic’, ‘quadriplegia’, and ‘quadriplegic’ June 2020 after institutional Research Ethics Board approval was obtained.

Both public and private online Facebook groups pertaining to SCI were searched. To access private groups, a standardized statement of intent was sent that included the study purpose, a declaration that no posts would be made, and that the group would be accessed for only 24 h. Facebook groups were excluded if they had: less than two members; access closed to individuals living with SCI; content not pertaining to SCI; non-English language or access denied for research purpose. Each included group was then searched for the terms ‘nerve transfer’, ‘nerve’, ‘transfer’, and ‘nerve surgery’. Data collected on each included group page included the group name, number of members, and number of times nerve transfer was stated. Each post about nerve transfer resulting from the described search terms, responses to these posts, and comments about nerve transfer in response to unrelated posts were tabulated. No personal identifiers of group members or post authors were recorded.

### Data analysis

Extracted data were analyzed using thematic content analysis by two authors (SM & JD).

Data were categorized into themes:

1. Seeking information: Posts requesting a description of personal experience with nerve transfer surgery, requesting objective information on nerve transfers, seeking a surgeon or center to perform the procedure, or asking for a second opinion.
2. Sharing information: Posts involving shared personal experience, giving objective information on nerve transfer surgery, describing alternative treatment, tagging a new person to share information, linking to outside resources, or recommending a specific surgeon or center.
3. Sharing support: Posts providing encouragement and condolences for other members without intent to share information.
4. Sharing appreciation: Posts thanking other members for their contribution.

## Results

**Fig. 1:**
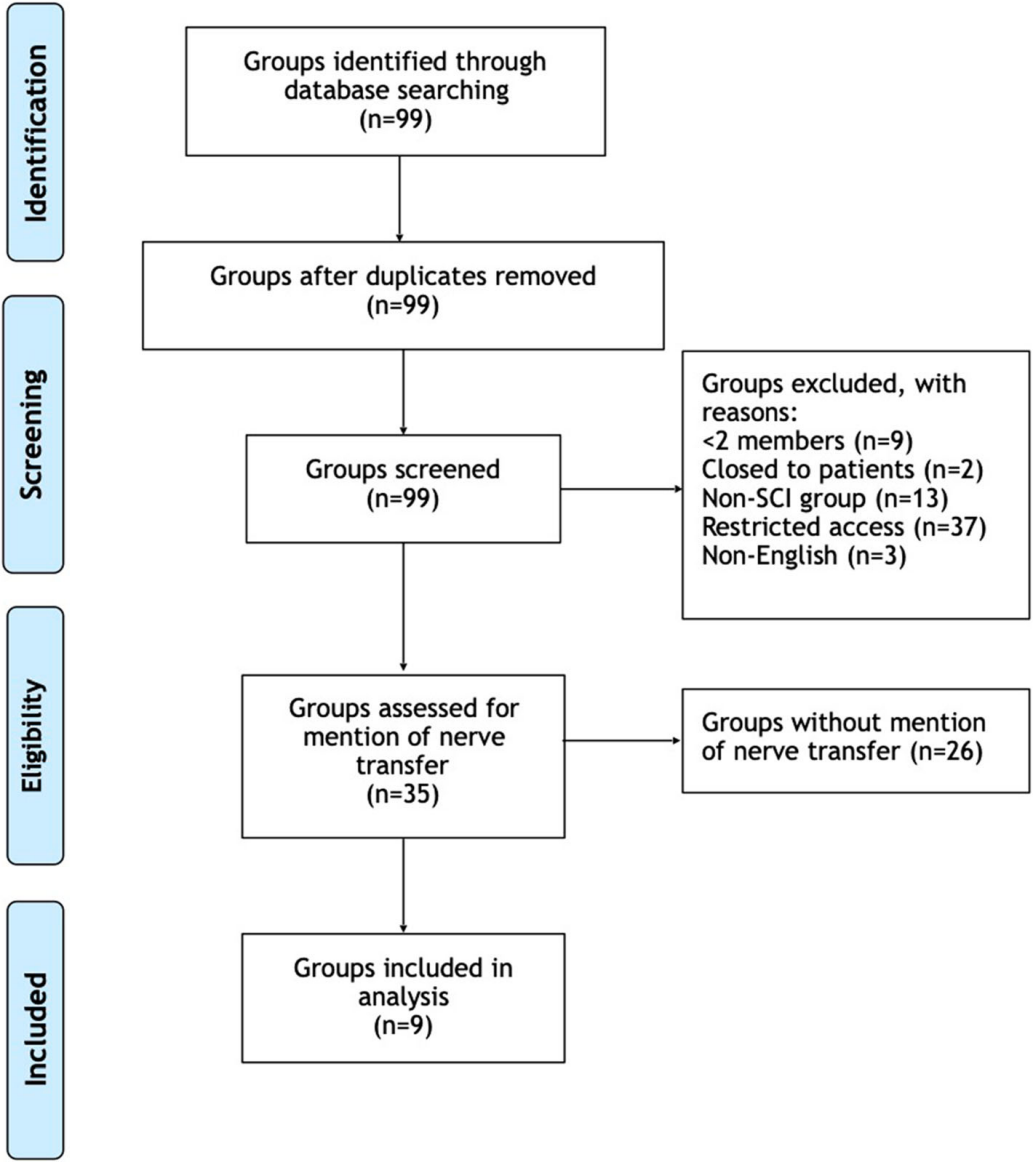
Search Flow Diagram. SCI = spinal cord injury

Our search yielded 99 Facebook groups, of which 35 were included in this study; 64 groups were excluded (Fig. 1). The average membership of included groups was 2007 (largest group = 12,277 members, smallest group = 8 members). Nerve transfer was discussed in nine groups, with 577 total mentions of nerve transfer and responses to nerve transfer posts (25% seeking information, 54% sharing information, 16% sharing support, 5% sharing appreciation). Of the 35 groups assessed for mention of nerve transfer, 23 were private and 12 were public. Of the 9 groups that screened positive for mention of nerve transfer and were included in this study, 5 were private and 4 were public.

In the seeking information axis, posts were related to seeking information on personal experience with nerve transfer (54%), objective information on nerve transfers (31%), surgeon/center to perform the procedure (9%), second opinions (4%), and alternative treatments (2%). Representative data points in this axis are presented in Table 1. At least 13% of posts seeking information were from individuals learning about nerve transfers for the first time.

**Table 1:** Seeking Information Axis: description, prevalence, and paraphrased representative quotes by theme

Seeking Information Axis	Axis Description	Prevalence, n (%)	Representative Quotes
**Personal experience**	Requesting individual narrative of first-hand experience of nerve transfer	76 (54%)	“Has anyone had any experience with nerve transfer in the hand, wrist or elbow to give function to the hands/fingers?”
**Objective information**	Seeking information on nerve transfer surgery, investigations, surgical candidacy, and rehabilitative process	44 (31%)	“So you mean nerve transfers follow a nerve study that shows results of peripheral nerve damage but must be done in first year?”
**Surgeon/center**	Requesting contacts for a qualified surgeon or a specific hospital where nerve transfer is performed	13 (9%)	“Would someone please private message me the surgeon that does this procedure?”
**Second opinion**	Seeking further information or alternative perspectives after a nerve transfer consultation	6 (4%)	“Do you have any negative feedback on nerve transfer? We have been interested in it but are afraid of having surgery, getting scars and then not having results. Thanks.”
**Alternatives**	Requesting information on treatments to restore upper extremity function outside of nerve transfer	3 (2%)	“Has anyone had Botox injections to affected finger? Did it work?”

In the sharing information axis, posts shared personal experience (52%), shared objective information on nerve transfer surgery (13%), linked to outside resources (12%), tagged a new person to share information with (11%), recommended a specific surgeon or center (9%; 1 plastic surgeon was recommended twice [2/27], 12/27 recommendations were for neurosurgeons, 4/27 for orthopaedic surgeons, and 9/27 recommendations were for specific centers), described alternative treatment (3%), and provided objective information on nerve injury (< 1%). Representative data points in this axis are presented in Table 2.

**Table 2:** Sharing Information Axis: description, prevalence, and paraphrased representative quotes by theme

Sharing information Axis	Description	Prevalence, n (%)	Representative Quotes
**Personal experience**	Individual narrative relaying experience of nerve transfer, or of injury and alternative treatment (i.e. tendon transfer) in direct response to a question regarding nerve transfer	162 (52%)	“My child had surgery last week with nerve grafts for both hands and tendon transfer on the right. In 6 months we expect enough strength to use a manual wheelchair, and in 2 months expect the ability to self-catheterize.”
**Objective information on nerve transfer**	Sharing information on nerve transfer surgery, investigations, surgical candidacy, and rehabilitative process	41 (13%)	“Typically neuro rehab or sports medicine doctors do electromyography. It isn’t invasive at all, takes place in regular patient rooms, and uses a very small needle and electrodes that send a small shock.”
**Link share**	Sharing links to externals sites with information on nerve transfer (i.e. news outlets and resources)	38 (12%)	“My partner participated in this study: https://www.bbc.com/news/health-48868670 ‘Rewiring nerves’ reverses hand and arm paralysis.”
**Tagging**	Mentioning the name of another group member in the comments to draw their attention to a post on nerve transfer	33 (11%)	N/A
**Surgeon/center**	Providing the name of a surgeon performing nerve transfer or a center where the procedure is performed	27 (9%)	“If there’s anyone looking for a good surgeon in USA, Dr. P in Atlanta does nerve and tendon transfers surgery! He has great communication and bedside manner and can help explain the procedure, and whether it’s an option for you.”
**Alternatives**	Sharing information on treatments to restore upper extremity function outside of nerve transfer	10 (3%)	“I’ve undergone stem cell treatments in Europe twice, both successful.”
**Objective information on nerve injury**	Providing information on the nature of nerve injury	3 (< 1%)	“It takes time for swelling on the spinal cord to go down. They will start to get motion back soon.”

Ninety comments in response to nerve transfer posts expressed support for the original post. A representative data point in the sharing support axis is “this is amazing! I’m praying that the outcome brings you the increased independence you are hoping for”. Thirty-one comments expressed appreciation for information shared on nerve transfer posts and responses. A representative data point in the sharing appreciation axis is “oh my, thank you so much! You are a shining light”.

## Discussion

Effective education on upper extremity reconstruction requires an understanding of the information accessible to the SCI community. Our study highlights a paucity of information on nerve transfer surgery available on social media. The information that is shared is of variable accuracy, and harbors biases based on the nature of personal experiences. Effective education strategies also require an understanding of how and what individuals with SCI wish to learn about their surgical options. The information sought by users in the seeking information axis here was primarily personal experience. These inferences can be used to guide future efforts to educate the SCI community on treatment options.

Nerve transfers can improve upper extremity function in persons with SCI with coaptation of a functioning motor nerve above the level of injury to a non-functioning recipient nerve [7]. Electrodiagnostic studies are used to determine candidacy for nerve transfer, and in people with intact lower motor neurons, surgery can improve function even decades after injury [7]. However, in individuals with a concomitant lower motor neuron injury [8], a limited duration of time to intervene exists [9, 10]. This makes timely knowledge of treatment options crucial for individuals with SCI who wish to improve upper extremity function.

People with SCI who pursue upper extremity reconstruction describe obtaining a substantial part of their knowledge about the procedure from peers who have undergone similar procedures [23]. This paradigm of personal learning highlights the role of social media platforms in connection and education. With increased functionality of the internet, there is a much broader network in which to find individuals with similar experiences and goals; this is particularly important for individuals with rare conditions. The internet also allows people the opportunity to learn from peers worldwide, whereas options were previously limited to the medical expertise in their circle of care. These benefits created by the internet are especially relevant to persons living with SCI, who may have mobility and geographic barriers to forming in-person social groups.

Despite the high level of engagement and large amount of health information available on social media, our Facebook search yielded little information on nerve transfer. Of the 35 groups searched, only 9 mentioned nerve transfer, and the greatest number of mentions within a single group was 336. This is a low number considering there were more than double this number of unique posts/comments (714) in this group on the day of the search alone, and the group has been active for almost six years, or 2130 days (with over 1 million posts). This is in keeping with a paucity of information on upper extremity reconstruction information found previously on the internet as a whole using the Google and Yahoo Bing search engines [16], and supports a need for more online information available on surgical options to improve upper extremity function. Within the context of our study, upper extremity surgery must be more accurately represented as a fundamental principle of tetraplegia care in online social forums.

The utility of peer-generated education on social media is limited by the accuracy of information shared. In general, information offered on Facebook showed good insight into the process and limitations of nerve transfers. Posts emphasized the role of pre-operative nerve conduction testing and electromyography, post-operative rehabilitation, and level of injury on possible outcomes from surgery. However, questions of what nerve transfer could achieve, or whether it was the best available treatment for an individual’s specific scenario, were not accurately answered. An experienced surgeon tailors each individual’s treatment to their specific injury pattern and treatment goals, using information on availability and quality of possible donors [24, 25]. Options for individuals are therefore highly variable, and this information cannot be easily shared.

Uncertainty around the quality of information provided on social media could limit uptake amongst individuals with SCI. The need for accurate, high-quality information on social media highlights the role of future collaboration between medical professionals and online communities. While it is essential to maintain the peer-based essence of these forums, there may be a benefit to physician verification of medical posts, guest appearance of surgeons for questions and answer sessions, or dissemination of posts or infographics prepared by a physician. Such collaboration has the potential to increase the accuracy and credibility of information available.

Personal experience may bias information being shared that is framed as general or objective. For example, a person who undergoes a nerve transfer by a surgeon from one surgical specialty may believe this is the only specialty that performs the procedure, which can propagate a false belief amongst the online SCI community. Of the 27 recommendations made for specialties and specialists offering nerve transfer, only two posts in our study recommended a plastic surgeon, despite plastic surgeons being on the forefront of nerve transfer surgery and peripheral nerve research [7, 13]. Similar biases can be introduced in the discussion of medical centers. In response to a post about a specific center performing nerve transfer, one commenter made plans to travel out-of-state to receive a consult, believing this was a unique opportunity unavailable elsewhere. While nerve transfer is a highly specialized surgery performed primarily at academic centers, it is available in many locations. Attempts to find a centralized list of centers offering nerve transfer yielded no results. This points to a gap in information that would be helpful to individuals seeking consultation for nerve transfer.

Despite the limitations of inaccuracies and biases in peer-generated information, personal experience was the most frequently sought form of information in our seeking information axis. This reflects a known proclivity to learn from peers as opposed to professionals or objective resources. Individuals who learn about upper extremity reconstruction from their primary care physician or physiatrist are less likely to proceed with surgery than those who learn about it from their peers [23, 26]. Compared to surgeons, physiatrists were found to be more comfortable offering nonoperative options [15], and less likely to identify the benefits of reconstruction [27]. An increase in peer-based learning may overcome this obstacle and lead more individuals to request referral to a surgeon for further information on reconstruction.

Questions in the seeking information axis also centered around gaining enough function to perform specific activities. Individuals pursuing reconstruction approach treatment education with personal goals, which often target recreation or employment [23]. This is in contrast to the measures of independence in activities of daily living, or the Medical Research Council (MRC) Scale for strength [12], that are frequently used outcome measures within the scientific literature [23]. While scales like the MRC offer benefits of validation and data that can be pooled for meta-analysis, they are inaccessible to a non-expert audience and may not reflect the outcomes most important to individuals considering undergoing reconstruction. This difference in perspectives and priorities between people living with SCI and physicians creates an obstacle when discussing both the individual value-add of pursuing reconstruction and the evidence supporting it. To bridge this gap, surgeons must consider the perspective of individuals living with SCI when framing reconstructive options within clinical discussion, as well as when designing studies on the topic.

This study highlights the need for increased awareness of nerve transfer and upper extremity reconstruction, and that social media is a prime platform for health information dissemination. Individuals living with SCI prefer information to be delivered by peers who use relatable language and experiences, and outcomes of interest must be considered from their perspective. These findings should inform future knowledge translation efforts to maximize education and subsequent uptake of upper extremity reconstructive surgery to improve function in cervical SCI. Specifically, strategies to incorporate expert knowledge with personal, peer-driven delivery on social media platforms must be developed. Ancillary to this effort, an information map of which surgeons and centers performing niche procedures should be made accessible.

### Limitations

Limitations of this study include use of a single online platform. Although Facebook is the dominant platform for health information exchange, other platforms including Twitter and YouTube have smaller but active health communities [17]. Instagram has also emerged as an important hub for persons living with SCI. Influencers in the SCI community with mass followings act to disseminate information. The substantial reach of these groups was not captured by our study, but does represent an avenue for future education campaigns. Additional limitations are exclusion of non-English groups, which may introduce selection bias, and inclusion of a single upper extremity reconstruction technique (nerve transfer) in the search strategy. We were specifically interested in discussion of nerve transfer because it is an emerging treatment with great potential impact, making it prime for mapping information dissemination patterns. In this way nerve transfer serves as a proxy for *how* information is exchanged about treatment options at large within the SCI community. Finally, conclusions may be limited by the accuracy of data collection, variations in care systems, and lack of independent verification.

## Conclusion

Nerve transfer can improve upper extremity function, which is a priority amongst people with cervical SCI. Despite this, both nerve transfer and other evidence-based upper extremity reconstruction options remain under-utilized. Lack of accessible and useful education on options contributes to this gap between evidence and practice. We identify social media as a source of information exchange among the SCI community. This platform is a potential target of future knowledge translation tools that combine a personalized media source with accurate, current medical messaging to optimize SCI education and increase upper extremity reconstruction uptake.

## Data Availability

The datasets analyzed during the current study are available from the corresponding author on reasonable request.

## Abbreviations

SCI: Spinal cord injury

## References

[1] Lude P, Kennedy P, Elfström ML, Ballert CS (2014). Quality of life in and after spinal cord injury rehabilitation: a longitudinal multicenter study. Top Spinal Cord Inj Rehabil.

[2] Manns PJ, Chad KE (2001). Components of quality of life for persons with a quadriplegic and paraplegic spinal cord injury. Qual Health Res.

[3] Putzke JD, Richards JS, Hicken BL, DeVivo MJ (2002). Predictors of life satisfaction: a spinal cord injury cohort study. Arch Phys Med Rehabil.

[4] Juguera Rodriguez L, Pardo Rios M, Leal Costa C, Castillo Hermoso M, Perez Alonso N, Diaz Agea JL (2018). Relatives of people with spinal cord injury: a qualitative study of caregivers’ metamorphosis. Spinal Cord.

[5] Snoek GJ, MJ IJ, Hermens HJ, Maxwell D, Biering-Sorensen F. (2004). Survey of the needs of patients with spinal cord injury: impact and priority for improvement in hand function in tetraplegics. Spinal Cord.

[6] Anderson KD (2004). Targeting recovery: priorities of the spinal cord-injured population. J Neurotrauma.

[7] Fox IK, Novak CB, Krauss EM (2018). The Use of Nerve Transfers to Restore Upper Extremity Function in Cervical Spinal Cord Injury. Pm r.

[8] Coulet B, Allieu Y, Chammas M (2002). Injured metamere and functional surgery of the tetraplegic upper limb. Hand Clin.

[9] Fu SY, Gordon T (1995). Contributing factors to poor functional recovery after delayed nerve repair: prolonged denervation. J Neurosci.

[10] Kobayashi J, Mackinnon SE, Watanabe O, Ball DJ, Ming Gu X, Hunter DA, et al. The effect of duration of muscle denervation on functional recovery in the rat model. Muscle Nerve. 1997;20(7):858–66. 10.1002/(SICI)1097-4598(199707)20:7<858::AID-MUS10>3.0.CO;2-O. 10.1002/(sici)1097-4598(199707)20:7<858::aid-mus10>3.0.co;2-o 9179158

[11] van Zyl N, Hill B, Cooper C, Hahn J, Galea MP (2019). Expanding traditional tendon-based techniques with nerve transfers for the restoration of upper limb function in tetraplegia: a prospective case series. Lancet..

[12] Khalifeh JM, Dibble CF, Van Voorhis A, et al. Nerve transfers in the upper extremity following cervical spinal cord injury. Part 2: preliminary results of a prospective clinical trial. J Neurosurg Spine. 2019:1–13. 10.3171/2019.4.SPINE19399. Online ahead of print. 10.3171/2019.4.SPINE19399 31299645

[13] Fox PM, Suarez P, Hentz VR, Curtin CM (2015). Access to surgical upper extremity care for people with tetraplegia: an international perspective. Spinal Cord.

[14] Curtin CM, Gater DR, Chung KC (2005). Upper extremity reconstruction in the tetraplegic population, a national epidemiologic study. J Hand Surg Am..

[15] Punj V, Curtin C (2016). Understanding and overcoming barriers to upper limb surgical reconstruction after tetraplegia: the need for interdisciplinary collaboration. Arch Phys Med Rehabil.

[16] Zhong S, Reed GE, Kalliainen LK. Upper Extremity Surgery in Tetraplegia and the Online Information Void. Hand (N Y)*.* 2019:1558944719878835. 10.1177/1558944719878835. Online ahead of print. 10.1177/1558944719878835 PMC8461191 31617411

[17] Canty MJ, Breitbart S, Siegel L, Fehlings D, Milo-Manson G, Alotaibi NM, Ibrahim GM (2019). The role of social media in selective dorsal rhizotomy for children: information sharing and social support. Childs Nerv Syst.

[18] Saxena RC, Lehmann AE, Hight AE, Darrow K, Remenschneider A, Kozin ED, Lee DJ (2015). Social media utilization in the cochlear implant community. J Am Acad Audiol.

[19] Shaw RJ, Johnson CM. Health Information Seeking and Social Media Use on the Internet among People with Diabetes. Online J Public Health Inform*.* 2011;3(1):ojphi.v3i1.3561. 10.5210/ojphi.v3i1.3561 PMC3615779 23569602

[20] Al Mamun M, Ibrahim HM, Turin TC (2015). Social media in communicating health information: an analysis of Facebook groups related to hypertension. Prev Chronic Dis.

[21] Beaudoin CE, Tao CC (2007). Benefiting from social capital in online support groups: an empirical study of cancer patients. CyberPsychol Behav.

[22] O'Riley AA, Rose J, Dalal B (2014). Online support for individuals with spinal cord injuries: an ethnographic investigation. J Spinal Cord Med..

[23] Dunn JA, Hay-Smith EJ, Keeling S, Sinnott KA (2016). Decision-making about upper limb tendon transfer surgery by people with tetraplegia for more than 10 years. Arch Phys Med Rehabil.

[24] Fox IK (2016). Nerve transfers in tetraplegia. Hand Clin.

[25] Fox IK, Miller AK, Curtin CM (2018). Nerve and tendon transfer surgery in cervical spinal cord injury: individualized choices to optimize function. Top Spinal Cord Inj Rehabil..

[26] Wagner JP, Curtin CM, Gater DR, Chung KC (2007). Perceptions of people with tetraplegia regarding surgery to improve upper-extremity function. J Hand Surg Am.

[27] Curtin CM, Wagner JP, Gater DR, Chung KC (2007). Opinions on the treatment of people with tetraplegia: contrasting perceptions of physiatrists and hand surgeons. J Spinal Cord Med.

